# Raptor/mTORC1 Acts as a Modulatory Center to Regulate Anti-bacterial Immune Response in Rockfish

**DOI:** 10.3389/fimmu.2019.02953

**Published:** 2019-12-18

**Authors:** Kang Li, Xiumei Wei, Libin Zhang, Heng Chi, Jialong Yang

**Affiliations:** ^1^State Key Laboratory of Estuarine and Coastal Research, School of Life Sciences, East China Normal University, Shanghai, China; ^2^Laboratory for Marine Biology and Biotechnology, Laboratory for Marine Ecology and Environmental Sciences, Qingdao National Laboratory for Marine Science and Technology, Qingdao, China; ^3^Key Laboratory of Marine Ecology and Environmental Sciences, Key Laboratory of Experimental Marine Biology, Institute of Oceanology, Chinese Academy of Sciences, Qingdao, China; ^4^Center for Ocean Mega-Science, Chinese Academy of Sciences, Qingdao, China

**Keywords:** teleost, mTORC1, raptor, lymphocyte, immune regulation, evolution

## Abstract

The mammalian target of rapamycin (mTOR) is an evolutionarily highly conserved atypical serine/threonine protein kinase, which regulates cell growth, proliferation, apoptosis, autophagy, and metabolism. As a regulatory protein, Raptor is awfully important for the stability and function of mTOR complex 1 (mTORC1). However, the studies about how Raptor/mTORC1 participates in and regulates immune response in lower vertebrates are still limited. In this study, we investigated the regulation of immune response by the Raptor/mTORC1 signaling pathway in rockfish *Sebastes schlegelii*. *Sebastes schlegelii* Raptor (Ss-Raptor) is a highly conserved protein during the evolution, in both primary and tertiary structure. Ss-Raptor mRNA was widely distributed in various tissues of rockfish and has a relative higher expression in spleen and blood. After infected by *Micrococcus luteus* or *Listonella anguillarum*, mRNA expression of Ss-Raptor rapidly increased within 48 h. Once Raptor/mTORC1 signaling was blocked by rapamycin, expression of the pro-inflammatory cytokines IL-1β and IL-8 was severely impaired, suggesting potential regulatory role of Raptor/mTORC1 signaling in the innate immune response of rockfish. In addition, Raptor/mTORC1 pathway participated in lymphocyte activation of rockfish through promoting 4EBP1 and S6 phosphorylation. Inhibition of Raptor/mTORC1 signaling crippled the lymphocyte expansion during primary adaptive immune response, manifesting by the decrease of lymphoid organ weight and lymphocyte numbers. More importantly, inhibition of Raptor/mTORC1 signaling impaired the lymphocyte mediated cytotoxic response, and made the fish more vulnerable to the bacterial infection. Together, our results suggested that Raptor and its tightly regulated mTORC1 signaling acts as modulatory center to regulate both innate and lymphocyte-mediated adaptive immune response during bacterial infection. This research has shed new light on regulatory mechanism of teleost immune response, and provide helpful evidences to understand the evolution of immune system.

## Introduction

The mammalian target of rapamycin (mTOR) is an evolutionary conserved serine/threonine protein kinase that belongs to the phosphatidylinositol 3-kinase-related kinase protein family (1). The mTOR pathway, which comprise mTOR and other several molecules, is one of the most important signaling that involved in numerous biological processes. It integrates multiple upstream signals such as energy, insulin, growth factors, and amino acids to regulate cell growth, proliferation, aging, movement, survival, protein synthesis, transcription, and metabolism (1–3). mTOR pathway is composed of two heteromeric complexes, mTORC1 and mTORC2 (4). Besides mTOR itself, mTORC1 consists of core protein, regulatory-associated protein of mTOR (Raptor), while mTORC2 contains core protein rapamycin-insensitive companion of mTOR (Rictor) (5–7). As an indispensable component of mTORC1, Raptor associated with mTOR, and could phosphorylate downstream S6K1 and 4EBP1 to initiate nucleic acid synthesis and cap-dependent translation (8), and phosphorylate SREBP1 et al. to promote cell metabolism (8–10). The activity of Raptor regulated mTORC1 signaling is highly sensitive to rapamycin treatment (9, 10).

As a pivot of signal transduction, mammalian mTOR signaling not only participates in vital biological processes, but also multiply regulated innate and adaptive immune response (11). Using a mouse model, scientists revealed that mTOR signaling plays an important role in the activation of T cells by integrating PI3K-AKT-mTORC1 pathway (12). mTORC1 determines the differentiation direction of Th1 and Th17 cells, and mTORC2 promotes the differentiation of Th2 cells (13). Meanwhile, mTOR pathway also regulates the inhibitory function of regulatory T cells (T_reg_) to maintain immune homeostasis (14), and performs as a mediator for T follicular helper cell (T_fh_) differentiation and germinal center formation (15). Additionally, mTOR signaling is also indispensable for the biological processes of a variety of innate immune cells, such as iNKT cell maturation, alveolar macrophage self-renewal, granulosa cells, and mast cell function, and antigen-presenting cells activation (16–18). As a binding protein of mTOR, Raptor regulates mTOR activity by binding to downstream substrates of mTOR, 4EBP1 and P70S6K. Deletion of Raptor impairs Th17 differentiation in a mouse model (19). Moreover, in intestinal epithelial, knockout of Raptor suppresses type 2 immune response during *Tritrichomonas muris* infection (20). Raptor also plays an instructive role in the development and function of B cells, and the deficient of Raptor impairs the survival and proliferation of pre-B cells (21). These progressive achievements suggest that mTOR signaling is a central hug to regulate both innate and adaptive immunity in mammals.

Although Raptor/mTORC1 pathway play important regulatory roles on mammalian immune system, knowledges about how this signaling controls immune responses of lower vertebrates is still limited. Recent studies have achieved great progresses regarding the regulation mechanism of innate immunity in teleost. Pattern recognition receptors (PRRs) recognizing pathogen-associated molecular patterns (PAMP), and thus initiating the downstream innate immune response are the approved strategy. Most PRRs presented in higher vertebrates have been found in teleost, such as Toll-like receptors (TLRs), RIG-I like receptors (RLRs), NOD-like receptors (NLRs), and C-type lectin receptors (CLRs) (22–24). Meanwhile, the interferon regulatory factors (IRF) family proteins regulate IFN gene expression via TLR, NF-κB, and other signals to trigger the innate immune response (25–27). microRNAs are also reported to serve as important positive or negative regulators in innate immunity of teleost by targeting different signaling proteins (28, 29). In addition, rainbow trout employs cathelicidin antimicrobial peptides as crucial components of the innate immune system to fight against pathogens (30, 31). On the other hand, the primitive vertebrate teleost has evolved lymphocytes mediated adaptive immune system. Receptors or molecules that peculiar to adaptive immunity, such as IgM/D/T, TCRα/β/γ/δ, MCHI/II, RAG1/2 and CD3/4/8, have been found to express in teleost leukocytes (32). CD8^+^ leukocytes from rainbow trout, freshwater carp and Atlantic salmon express high level of cytotoxicity-related genes, such as perforin A, granzyme B and IFN-γ, after antigen or pathogen stimulation, indicating their potential similar properties with CD8^+^ T cells in mammals (33, 34). Different with mammalian, teleost B cells express three different kinds of immunoglobulins, IgM, IgD and IgT, and these immunoglobulins seem to play immunological roles in different locations (35, 35–37). However, compared with innate immunity, the regulatory mechanism of adaptive immunity remains largely unknown in teleost. In our previous study, two components of MAPK/ERK pathway, h-Ras and c-Raf, were reported to control adaptive immune response of Nile tilapia by regulating lymphocyte activation (38, 39). Here, we identified a Raptor homolog from rockfish *Sebastes schlegelii*. After analyzing its sequence, structure, phylogenetic, and expression characteristic, the potential roles of Raptor-regulated mTORC1 signaling on rockfish innate and adaptive immune response were investigated. The aim of present study is to provide theoretical proofs for the regulation of Raptor/mTORC1 on lymphocyte-mediated immune responses in teleost, and to shed a novel perspective for the evolution of immune system.

## Materials and Methods

### Experimental Animals

Rockfish ordered from an aquatic farm in Yantai, Shandong province, China were raised in aerated seawater at 15°C. Fish were fed with commercial pellets daily, and health fish with body length of 8–10 cm were used for experiments.

### Sequence and Structural Analyses

We obtained the cDNA sequence of Ss-Raptor from the previous transcriptome library we constructed, and submitted it to NCBI GenBank with an accession number of MK801672. The amino acid sequences of Raptor and homologous proteins of other species were analyzed using the NCBI Protein BLAST. The multiple sequence alignment was analyzed with the software ClustalX 1.83 and displayed by the sequence manipulation suite (SMS). The phylogenetic tree was constructed by MEGA5 through neighbor joining (NJ) algorithm. The main protein domains were predicted by the simple modular architecture research tool (SMART) and NCBI Protein BLAST, and was laid out by DOG. We established the tertiary structures of proteins with SWISS-MODEL prediction algorithm and displayed them by PyMOL software.

### mRNA Expression of Ss-Raptor in Different Tissues

Blood were collected from caudal vein with anticoagulant (0.45 M NaCl, 0.015 M sodium citrate, 0.1 M glucose, and 0.01 M EDTA, pH 7.0) and centrifuged at 4,000 rpm, 4°C for 5 min to settle the cells. The total RNA of blood, head kidney, muscle, liver, blood, spleen, gill, or trunk kidney were extracted from four random healthy rockfish with TRIzol reagent (Invitrogen). The total RNA was treated with DNase at 37°C for 30 min to degrade the remaining DNA, and at 65°C for 10 min to stop reaction. Then, the obtained RNA was used as templates to synthesize the first strand cDNA according to the M-MLV reverse transcriptase product information sheet (Promega). The 1:30 diluted cDNA was used as the template for SYBR Green fluorescent real-time quantitative PCR (qPCR). β-actin was used as internal control. The gene-specific primers for Ss-Raptor and β-actin were listed in Table 1. Expression level of genes was analyzed by comparative Ct method (2^−ΔΔCt^ method).

**Table 1:** qPCR primers used in the present study.

**Primer**	**Sequence (5^**′**^-3^**′**^)**
Ss-β-actin (forward)	GCATCACACCTTCTACAACGAGC
Ss-β-actin (reverse)	TCTTCTCCCTGTTGGCTTTGG
Ss-Raptor (forward)	ACTGAGACGCAACGCCAAAG
Ss-Raptor (reverse)	GACTTGACGATGATTCCCGC
Ss-IL-1β (forward)	CGAGGGACTGGACTTTGAGATTTC
Ss- IL-1β (reverse)	CAGCATGATGTTGAGCAGGTCTTC
Ss-IL-8 (forward)	TCTATTGTGGTGCTCCTGGCTTTC
Ss-IL-8 (reverse)	AATGGGAGTTGGCAGGAATCAG
Ss-TNFα (forward)	ACGATACAGCCTGACAGCCATTC
Ss-TNFα (reverse)	TGGAGTAGTTGAAACCGCCTTGAC
Ss-Granzyme B (forward)	ACAGGTATGTGAAGGTCATCGGA
Ss- Granzyme B (reverse)	CATAGCATGTGAGCAGGATTGAAG
Ss-Perforin A (forward)	GGATGAAGACAACAAATGGGACGA
Ss- Perforin A (reverse)	ATGAGCATGGCGAGACACATACAG

### Bacterial Challenge and Rapamycin Treatment

The strain of *Listonella anguillarum* was kindly gifted by Dr. Jianmin Zhao from Yantai Institute of Coastal Zone Research, Chinese Academy of Sciences. For temporal expression of Ss-raptor after bacterial infection, healthy rockfish was immersed in 1 × 10^7^ CFU mL^−1^ *Micrococcus luteus, L. anguillarum* suspension or left unstimulated. Head-kidney or blood were harvested from four random fish at 0, 6, 12, 24, and 48 h after challenge for qPCR analysis.

For rapamycin inhibition during microbe infection, healthy rockfish that intraperitoneally (*i.p*.) injected with 1 × 10^5^ CFU mL^−1^ *L. anguillarum* suspension in PBS was *i.p*. injected with 75 μg/kg of rapamycin on day 1, 2, 3, and 5 days, and rockfish injected with the same volume of PBS was used as control. The head-kidney was harvested on day 2 and day 7 post infection for pro-inflammatory cytokine and cytotoxic gene expression assay by qPCR, respectively. The gene-specific primers used qPCR were listed in Table 1. The liver was harvested on day 7 after infection and fixed with Bouin's fluid for 24 h, then transferred to 70% alcohol solution for H&E staining. For survival analysis, rockfish with or without rapamycin treatment as above was *i.p*. injected 1 × 10^5^ CFU mL^−1^ *L. anguillarum* suspension, and fish mortality during infection was recorded and calculated.

### Isolation of Lymphocytes

Spleen and head-kidney lymphocytes were isolated from rockfish using Lymphocyte Separation Medium (TBD, China). Briefly, spleen and head-kidney were smashed in Leibovitz's L-15 medium (Gibco) and collected through a cell filter membrane. The cell suspension was centrifuged at 250 g, 4°C for 5 min and the cell pellet was resuspended in 5 mL L-15 medium prepared on ice. Then, the cell suspension was added to the centrifuge tube with 5 mL separation medium and kept stratified, then centrifuged at 500 g, room temperature for 30 min with the lowest acceleration and deceleration. The second layer of the ring-shaped cells was carefully pipetted into another centrifuge tube, washed twice with L-15 medium for subsequent experiments.

### Giemsa Staining and H and E Staining

For Giemsa staining, resuspended cells were centrifuged onto a slide at 1,000 rpm for 3 min using Cytospin 4, and the cells were fixed in methanol for 10 min. The cells were stained with Giemsa dye solution (BBI Life Sciences) for 30 min. After washing three times with PBS, the sample is sealed with a neutral resin. For H&E staining, liver was prepared in Bonn solution for 24 h followed by dehydration in 70, 80, 90, 95, and 100% ethanol. After be transparented in xylene, the dehydrated sample was transferred to paraffin wax. Obtained sample was embedded in paraffin and sectioned (7 μm). Hematoxylin staining was performed after the samples were treated with xylene-xylene-100% ethanol-100% ethanol-95% ethanol-80% ethanol. Subsequently, the sections were counterstained with eosin and washed with water, and followed by the treatment with 80% ethanol-90% ethanol-95% ethanol-100% ethanol-100% ethanol-xylene-xylene, before they were sealed with a neutral resin. The cell and tissue samples were observed by a light microscope (Olympus).

### Lymphocyte Stimulation

The spleen lymphocytes were resuspended in 500 μL D-PBS (with Ca^2+^ and Mg^2+^, BBI Life Sciences), rested at 20°C for 30 min to remove innate phosphorylation, and then treated with 50 ng mL^−1^ phorbol 12-myristate 13-acetate (PMA, Sigma) plus 500 ng mL^−1^ ionomycin. The stimulated lymphocytes were collected at 5, 15, and 45 min, respectively, and 500 μL pre-cooled D-PBS was added to terminate stimulation. The rested lymphocytes were used as control. The collected cells were used for western-blot analysis.

### Western-Blot Analysis

After spin down, the stimulated cells were resuspended in NP40 lysis buffer (50 mM Tris-HCl, 150 mM NaCl, 1% NP-40) on ice for 30 min, and centrifuged at 13,000 g, 4°C for 10 min to gain supernatant. The protein samples were separated by SDS-PAGE and transferred onto a nitrocellulose (NC) membrane in a waterish electrophoretic transfer system at 100 V for 120 min. The membrane was blocked with 4% non-fat powdered milk at room temperature for 1 h, and then incubated with 1:1,000 diluted rabbit anti-AKT, anti-S6, anti-4EBP1, anti-phospho-AKT (Thr308), anti-phospho-S6 (Ser240/244), anti-phospho-4EBP1 (Thr37/46), or anti-β-actin (Cell Signaling Technology) in 2% bovine serum albumin at 4°C overnight, respectively. After three rinses with PBST (PBS with 0.05% tween-20), the membrane was incubated with goat anti-rabbit IgG H&L Alexa Fluor 790 (Abcam, 1:10,000) in 4% non-fat powdered milk at room temperature for 1 h. The membrane was scaned by Odyssey CLx Image Studio after another three rinses. The β-actin was selected as internal control.

### Immunofluorescence Assay

Spleen lymphocytes that stimulated with PMA plus ionomycin for 15 min were spin onto slides with Cytospin 4, and resting lymphocytes were used as control. After fixed in methyl alcohol for 5 min, the slides were blocked with 1% BSA at room temperature for 1 h. The lymphocytes were then incubated with rabbit anti-p-4EBP1(Thr37/46) (1:200 dilution) and mouse anti-β-actin (1:2,400) antibodies at 37°C for 1 h. Cells were incubated with Alexa Fluor 488-conjugated goat anti-rabbit IgG (H + L) and Alexa Fluor 594-conjugated goat anti-mouse IgG (H + L) (1:1,000 dilution) at 37°C for another 1 h. After each incubation, a thrice rinse with PBST was performed. The slides were mounted with sealing glycerol containing Hoechst-33342 and finally observed with a fluorescence microscope (Olympus).

### Statistical Analysis

All data were displayed in terms of mean ± SE. The 2-tailed Student's *t*-test was applied to define the statistical significance. The *p* < 0.05 was labeled with ^*^, <0.01 with ^**^, and <0.001 with ^***^ above the bars in figure.

## Results

### Sequence Characteristics of Ss-Raptor

The full length of Ss-Raptor cDNA is submitted to NCBI GenBank with accession number of MK801672. It is 5,314 bp, consisting of a 445 bp 5'UTR, an 816 bp 3' UTR and a 4053 bp CDS. The CDS encodes a protein of 1,350 amino acids with the predicted molecular weight of 151.25 kDa and theoretical isoelectric point of 7.01. According to NCBI Protein Blast, Ss-Raptor protein shares more than 98% identity with Raptor from *Larimichthys crocea* (XP_010732413.1), *Seriola lalandi dorsalis* (XP_023262644.1), and *Acanthochromis polyacanthus* (XP_022048743.1).

**Figure 1:**
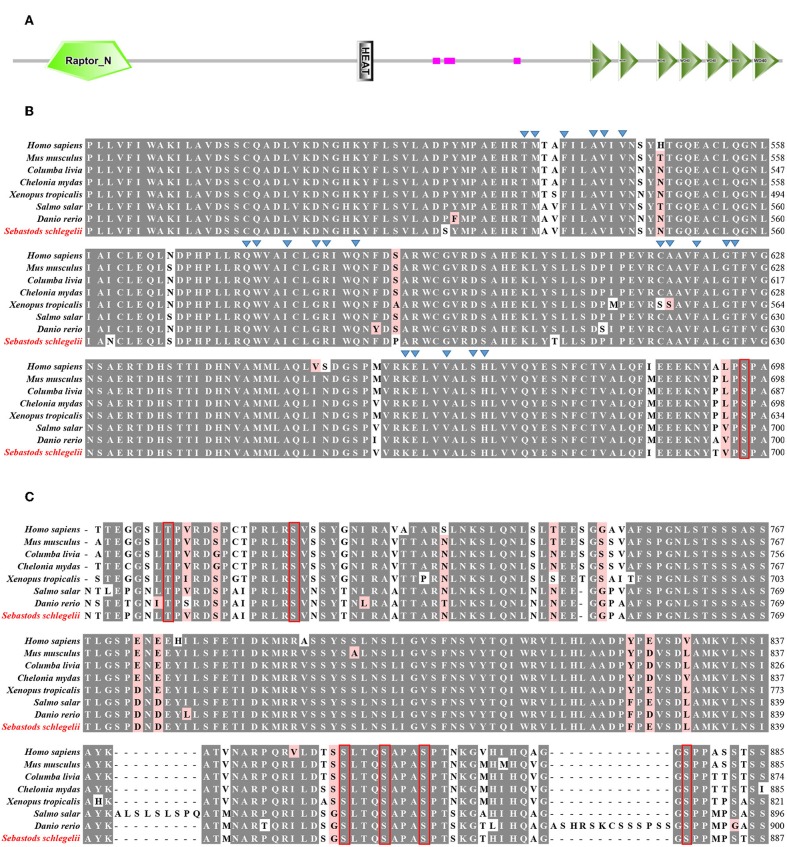
Sequence alignment of Ss-Raptor with its homologs from other species. **(A)** The structure of Raptor protein in rockfish by SMART prediction. **(B)** Multiple sequence alignment analyses of Heat repeat domain in Raptor. Peptide binding sites are indicated with triangle, and the conservative phosphorylation point is shown in red box. **(C)** Multiple sequence alignment analyses of phosphorylation sites between Heat repeat domain and WD40 domain of Raptor. The conserved phosphorylation point is shown in red box. The background of the amino acid residues conserved in 80% are shown as dark gray and the similar residues is light red. The selected protein sequences are *Homo sapiens* (NP_065812.1), *Mus musculus* (NP_065812.1), *Columba livia* (PKK18984.1), *Chelonia mydas* (XP_007056109.1), Xenopus tropicalis (XP_012827010.1), *Salmo salar* (XP_014032262.1), *Danio rerio* (XP_021327417.1).

According to SMART prediction, amino acid sequence of Ss-Raptor is composed of a Raptor N-terminal conserved domain (RNC), a heat repeat domain that is associated with interactions between proteins, and seven tandem WD40 domain in the C-terminal that are also responsible for interactions between proteins (Figure 1A). The amino acid sequence of Ss-Raptor, especially the HEAT repeat domain (Figure 1B) and WD40 domain (Figure 1C), was highly conserved with the homologs from other vertebrates. More importantly, functional motifs, such as putative peptide binding sites in HEAT repeat domain, and two phosphorylation site clusters in WD40 domain, were almost the same in all the selected Raptors (Figures 1B,C), suggesting the potential conservation of this molecule during the species evolution.

### Structural Character of Ss-Raptor

**Figure 2:**
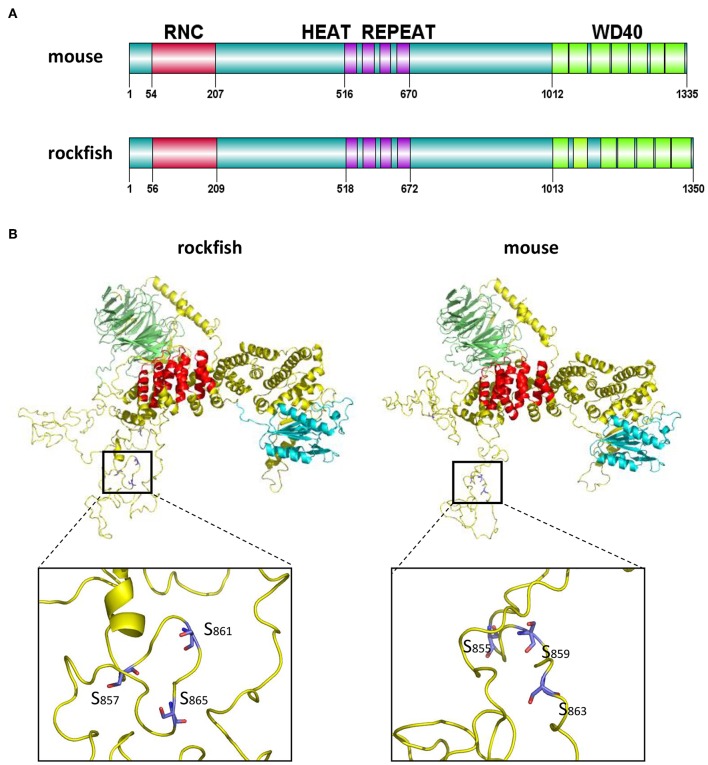
The domains and tertiary structure analysis of Ss-Raptor. **(A)** Comparison of domains organization between Raptors from mouse (NP_065812.1) and rockfish. **(B)** Comparison of the three-dimensional structure between Raptor proteins from mouse and rockfish by SWISS-MODEL. Different colors represent different structural domains (green, WD40 domain;red, Heat repeat; cyan, RNC; yellow, unstructured domain). The position of conserved phosphorylation sites in the spatial structure was highlighted.

Compared with Raptor in mouse, Ss-Raptor employs a highly similar way to organize its functional domains (Figure 2A). Furthermore, the three-dimensional structure predicted by SWISS MODLE suggests that, the 3D structures of Raptor from rockfish and mouse share high spatial similarity in the main structural domains and the overall conformation (Figure 2B). In addition, the spatial position of several key phosphorylation sites, for example S855, S859, S863 in mouse and S857, S861, S865 in rockfish, are also well conserved (Figure 2B). Thus, rockfish Raptor shares highly similarity of structure with its homolog from mammal, suggesting the potential similar functions it may performed with that in higher vertebrates.

### Phylogenetic Position of Ss-Raptor

**Figure 3:**
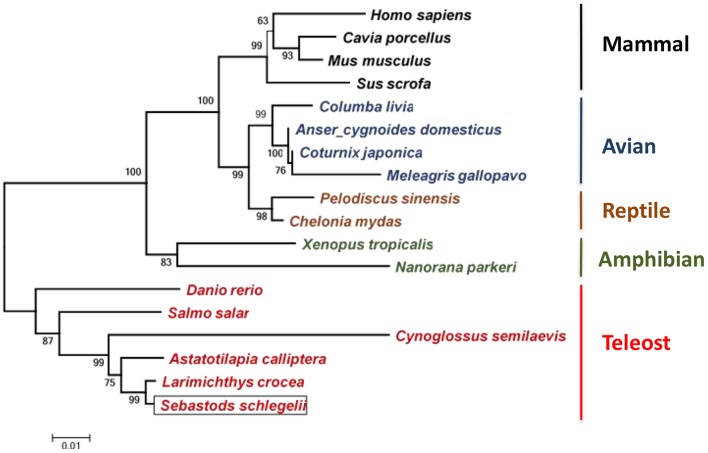
Phylogenetic tree of Raptors from different vertebrates. Species clustered well are marked with the same color. The selected protein sequences are *Homo sapiens* (NP_065812.1), *Mus musculus* (NP_065812.1), *Cavia porcellus* (XP_003464843), *Sus scrofa* (JAA53799.1), *Columba livia* (PKK18984.1), *Meleagris gallopavo* (XP_016210883), *Coturnix japonica* (XP_015735506.1), *Anser cygnoides domesticus* (XP_013053394.1), *Pelodiscus sinensis* (XP_006119416.1), *Chelonia mydas* (XP_007056109.1), *Salmo salar* (XP_014032262.1), *Danio rerio* (XP_021327417.1), *Larimichthys crocea* (XP_010732413.1), *Astatotilapia calliptera* (XP_026025586.1), *Cynoglossus semilaevis* (XP_024914266.1), *Xenopus tropicalis* (NP_001123843.1), *Nanorana parkeri* (XP_018422015). Bootstrap values of 1,000 replicates (%) are indicated for the branches.

The amino acid sequences of Ss-Raptor and other Raptors from different vertebrates were collected to construct the phylogenetic tree using neighbor joining algorithm based on multiple sequence alignment. The Ss-Raptor firstly clusters with Raptor from *Larimichthys crocea*, and then clusters with the same molecules from other teleost (Figure 3), suggesting a close relationship of Ss-Raptor with its homologs from teleost. The Raptors from other vertebrates, such as amphibian, avian, reptile and mammal, form sister groups of the group formed by teleost Raptors (Figure 3). These observations suggested that Raptor is a highly conserved molecule from fish to mammals throughout the vertebrate evolution.

### mRNA Expression Pattern of Ss-Raptor in Different Tissues

**Figure 4:**
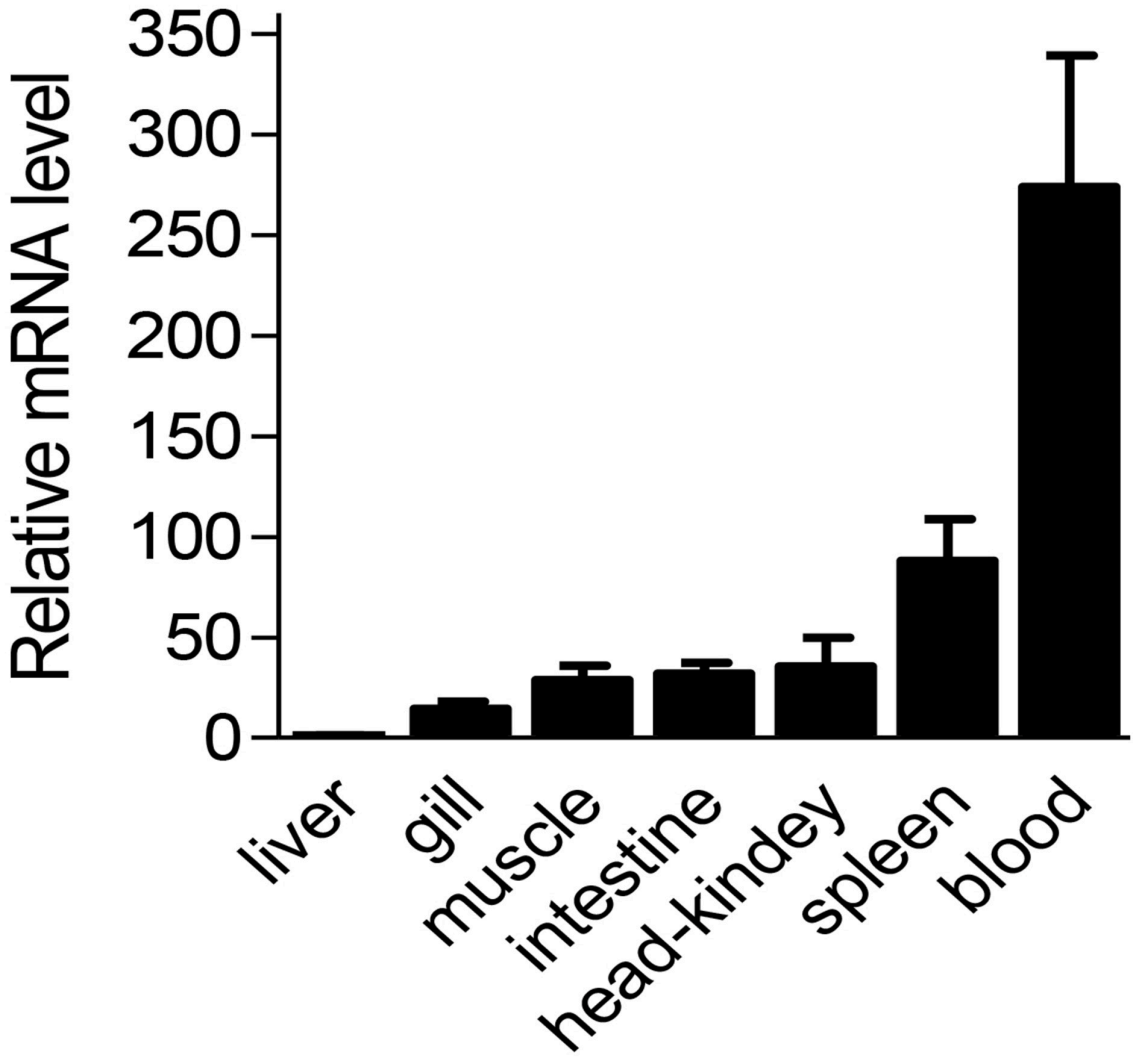
The mRNA level of Ss-Raptor in different tissues detected by qPCR. Ss-Raptor expression levels in blood, spleen, gill, muscle, intestine and head-kidney were normalized to that in liver. The vertical bars represent mean ± SE (*n* = 4).

Considering the high conservation of Ss-Raptor in sequence, structure and phylogeny, we suspect it might perform similar regulatory function to immune system of rockfish as those in higher vertebrates. We initiated our functional studies by determining mRNA expression pattern of Ss-Raptor in different tissues. The result of qPCR assay showed that Ss-Raptor mRNA was widely expressed in all the tested tissues, including liver, gill, muscle, intestine, head-kidney, spleen and blood (Figure 4). Among these tissues, the expression level of Ss-Raptor mRNA was highest in blood, which was 253-fold than that in liver (Figure 4). Meanwhile, Ss-Raptor mRNA also expressed with higher level in head-kidney and spleen, which was 29- and 81-fold of that in liver, respectively (Figure 4). Other tissues, such as gill, muscle, and intestine expressed moderate Ss-Raptor levels, and Ss-Raptor mRNA expressed the lowest in liver (Figure 4). The relative higher expression level of Ss-Raptor in lymphoid related tissues, such as blood, spleen and head-kidney, indicated a potential participation of Ss-Raptor in immune response.

### Rockfish Raptor/mTORC1 Regulates Innate Immunity During Anti-bacterial Response

**Figure 5:**
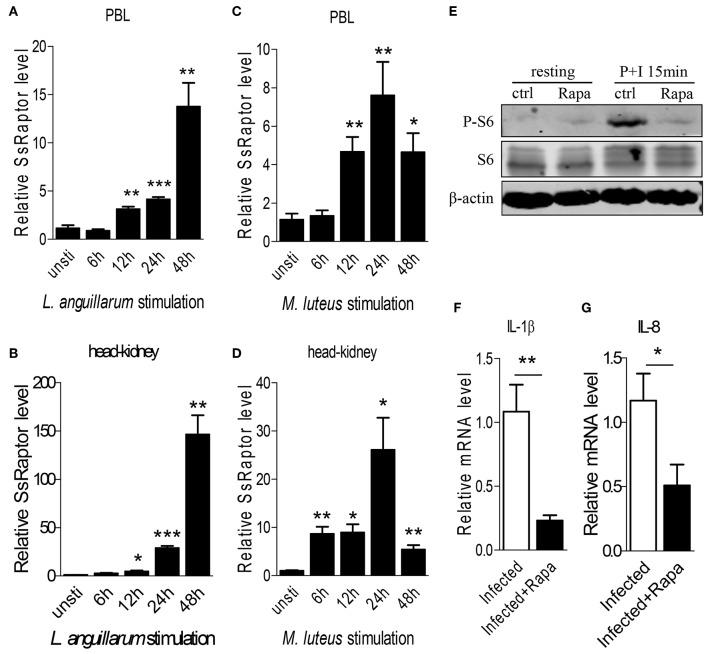
Ss-Raptor regulates innate immune response during bacterial infection. **(A–D)** Rockfish was infected with *L. anguillarum* **(A,B)** or *M.luteus* **(C,D)**, mRNA expression of Ss-Raptor in PBL **(A,C)** and head-kidney **(B,D)** was examined by qPCR at 6, 12, 24, and 48 h post stimulation, *n* = 4. **(E)** Spleen lymphocytes untreated or treated with PMA plus ionomycin were analyzed by immunoblot analysis with antibodies as indicated. **(F,G)** Rockfish was untreated or treated with rapamycin after *L.anguillarum* stimulation. The mRNA level of IL-1β **(F)** and IL-8 **(G)** were detected by qPCR (*n* = 4) *p < 0.05, **p < 0.01, ***p < 0.001.

To figure out the involvement of Ss-Raptor in innate immune response of rockfish, we detected the mRNA expression of Ss-Raptor during *M. luteus* or *L. anguillarum* infection. Transcription level of Ss-Raptor, both in blood and head-kidney, was significantly induced within 48 h after *M. luteus* or *L. anguillarum* infection (Figures 5A–D). After Gram-negative bacteria *L. anguillarum* stimulation, Ss-Raptor mRNA began to upregulate at 12 h and reached the peak at 48 h (Figures 5A,B). While the mRNA of Ss-Raptor reached the maximum expression level at 24 h, and began to decrease at 48 h post stimulation, however which still remained at a higher level compared with that in control group (Figures 5C,D). The intensely inducible expression of Ss-Raptor in the innate immune stage suggests the potential involvement of Raptor in the anti-bacterial innate immune response of rockfish.

To further investigate the regulatory role of Raptor/mTORC1 signaling on innate immune response of rockfish, we treated the animals with inhibitor rapamycin to block Raptor/mTORC1 signaling. Treatment of rapamycin severely impaired the PMA plus ionomycin-induced S6 phosphorylation (Figure 5E), suggesting the effective inhibition of mTORC1 signaling by rapamycin. Since *L. anguillarum* is a potential pathogen for marine fish, and it cause relative stronger up-regulation of SsRaptor than *M. luteus* (Figures 5A–D), we thus selected *L. anguillarum* as an bacteria example for the following study. During *L. anguillarum* infection, we suppressed the Raptor/mTORC1 signaling, and examined the mRNA expression of two pro-inflammatory cytokines, IL-1β and IL-8, by qPCR. Inhibition of mTORC1 activity severely impaired the expression level of both IL-1β and IL-8 (Figures 5F,G). Overall, these data indicate that the Raptor and its regulated mTORC1 signaling are involved in, and play a crucial role in the anti-bacterial innate immune response of rockfish.

### Raptor/mTORC1 Participates in Lymphocytes Activation of Rockfish

**Figure 6:**
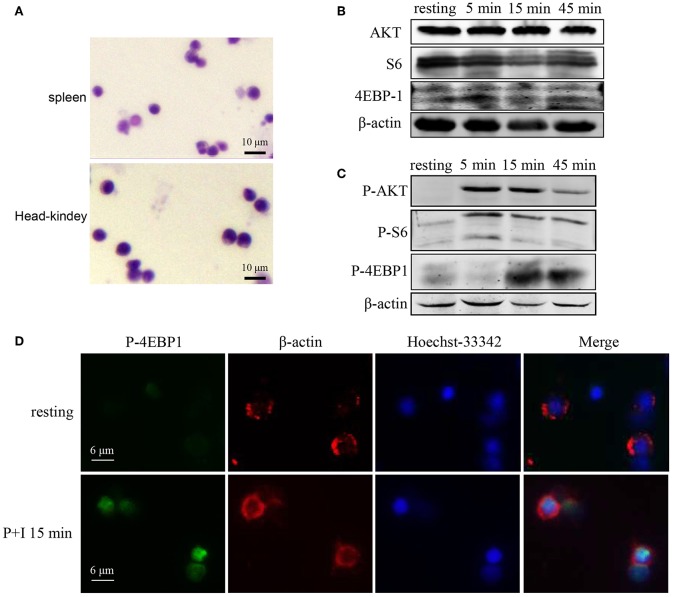
Raptor/mTORC1 axis is involved in the lymphocyte activation of rockfish. **(A)** The morphology of isolated lymphocytes was observed by microscope after Giemsa staining. **(B,C)** Spleen lymphocytes untreated or treated with PMA plus ionomycin for different time points were analyzed by immunoblot analysis with antibodies as indicated. **(D)** The phosphorylation level of 4EBP1 in spleen lymphocytes which untreated or treated with PMA plus ionomycin for 15 min was detected by Immunofluorescence analysis.

Next, we sought to know whether Raptor/mTORC1 signaling is associated with adaptive immunity of teleost. Considering lymphocytes are the main executant of adaptive immunity, we first investigated the participation of Raptor/mTORC1 signaling in the lymphocyte activation of rockfish. The cells isolated from rockfish spleen and head-kidney using Lymphocyte Separation Medium were round and with a high nucleus/cytoplasm ratio (Figure 6A), which were consistent with typical lymphocyte morphology. Then, PMA plus ionomycin were used to activate lymphocyte receptor signaling *in vitro*, and the protein and phosphorylation level of key components in mTORC1 signaling was examined by western-blot and immunofluorescence. After lymphocyte activation, although there was no obvious elevation of AKT, S6 or 4EBP-1 at total protein level (Figure 6B), phosphorylation of AKT (Thr308) that upstream of mTORC1, S6 (Ser240/244), and 4EBP1 (Thr37/46) that downstream of mTORC1 were promptly and intensely enhanced (Figure 6C). Meanwhile, the augment of 4EBP1 phosphorylation was also confirmed by immunofluorescence at 15 min after lymphocytes was stimulated by PMA and ionomycin (Figure 6D). These results suggest that Raptor/mTORC1 signaling activation is an important event that associated with lymphocyte activation in rockfish.

### Raptor/mTORC1 Is Important for Rockfish Lymphocyte Expansion

**Figure 7:**
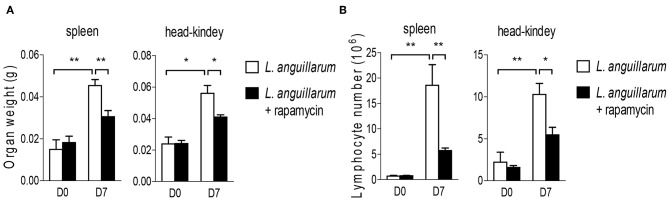
Raptor/mTORC1 axis regulates lymphocyte expansion in rockfish. Rockfish was untreated or treated with rapamycin after *L.anguillarum* stimulation. **(A)** The weight of lymphoid organs was measured on day 7 post infection (*n* = 7). **(B)** The absolute number of lymphocytes within spleen and head-kidney were measured by blood counting chamber on day 7 post infection (*n* = 7) *p < 0.05, **p < 0.01.

After activation, lymphocytes proliferate promptly to exert effector function, and mTORC1 signaling is crucial for lymphocytes expansion in mammals. Therefore, we sought to determine whether the Raptor/mTORC1 also play a certain role in the lymphocyte proliferation of rockfish during bacterial infection. Since it takes 6–8 days for lymphocytes to expand and reach the peak, this timepoint is usually used for studying lymphocyte-mediated primary immune response in mammals (40). And this window phase is also fit for teleost adaptive immune response (41). *L. anguillarum* infected rockfish was treated with rapamycin to inhibit mTORC1 activity or not. Seven days after infection, the weight of lymphoid organs, both spleen and head-kidney, was obviously increased compared with uninfected controls (Figure 7A). However, after mTORC1 blockade by rapamycin, the augment of lymphoid organs' weight was significantly impaired than that in the animals that without rapamycin treatment (Figure 7A). Coincidentally, the total lymphocyte numbers, in both spleen and head kidney, were dramatically increased on day 7 after *L. anguillarum* infection, and the robust expansion of lymphocytes was severely impaired once mTORC1 signaling was suppressed by rapamycin (Figure 7B). Therefore, our results that inhibition of mTORC1 by rapamycin impairs the lymphoid organs weight and lymphocyte number during adaptive immune response, suggest that rockfish Raptor/mTORC1 signaling promotes pathogen-induced lymphocytes expansion.

### Raptor/mTORC1 Is Indispensable for Lymphocytes to Eliminate Infection

**Figure 8:**
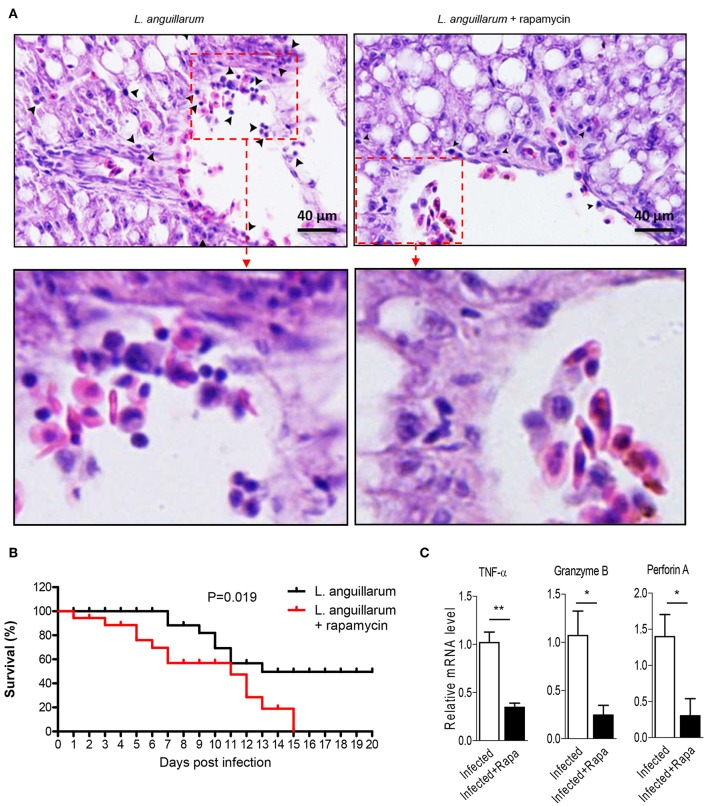
Raptor/mTORC1 axis is indispensable for rockfish lymphocytes to eliminate infection. Rockfish was untreated or treated with rapamycin after *L.anguillarum* stimulation. **(A)** HE staining shown the inflammatory cells infiltration in rockfish liver on day 7 after infection. **(B)** The survival percentage of animals untreated or treated with rapamycin after infection was shown (*n* = 30). **(C)** The mRNA level of TNF-α, Granzyme B, and Perforin A in spleen lymphocytes were detected by qPCR on day 7 after infection (*n* = 4). *p < 0.05, **p < 0.01.

Lymphocytes eliminate pathogen infection by releasing a series of cytokines and inducing inflammatory response. In this study, we investigated the potential regulatory role of Raptor/mTORC1 signaling on lymphocytes mediated effector function. During *L. anguillarum* infection, rockfish was treated with rapamycin to inhibit mTORC1 activity. Blockade of mTORC1 signaling impaired the pathogen-induced inflammatory infiltration in the liver 7 days after infection (Figure 8A). Correlated with that, lack of mTORC1 activity made the fish more vulnerable to pathogen infection, revealing by the higher death rate of rockfish that treated with rapamycin than un-inhibited control within 15 days (Figure 8B). Moreover, mRNA expression levels of important genes that highly associated with cytotoxic ability with effector T cells, such as TNF-α, Granzyme B and perforin A, were obviously decreased compared with their corresponding controls (Figure 8C). Thus, these results suggest that Raptor/mTORC1 signaling is required for rockfish effector lymphocytes to eliminate bacterial infection during adaptive immune response.

## Discussion

Benefiting from its prominent ability to sense and integrate immune signals, Raptor/mTORC1 pathway plays central roles to ensure the proper function of immune system (42). Nevertheless, the vast majority of studies are focusing on higher vertebrates, especially the mammals, whether and how this signaling control immune responses in early vertebrates remains largely unknown. Herein, using rockfish as a model, we investigated the participation and potential regulatory mechanism of Raptor/mTORC1 signaling on innate and adaptive immunity of teleost.

As a key scaffold protein of mTORC1, Raptor is involved in the regulation of immune response. Like its homologs in human and mouse, Ss-Raptor comprises of three conservative domains, including an RNC domain, four Heat Repeat domain and seven WD40 domain (43). Heat repeat domain appear to function as protein-protein interaction surfaces and is widely present in cytoplasmic proteins such as TAF6. Deletion of the Heat repeat domain severely impairs transcriptional activation of TAF6 and interactions with other proteins (44). mTOR also contains conserved Heat repeat domain which forms a stable scaffold that mediates interactions with upstream and downstream protein (45). In addition, repeated WD40 motifs act as a site for protein-protein interaction, and proteins containing WD40 repeats are known to serve as platforms for the assembly of protein complexes. Moreover, the predicted polypeptide binding sites in Heat Repeat domain, which are crucial important for protein-protein interaction, are also highly conserved. Therefore, we deduce that the WD40 and Heat repeat domains are pivotal for Raptor to interact with mTOR and its downstream substrates in rockfish. Raptor phosphorylation is crucial for Raptor-mTOR interaction and mTOR activity, meanwhile mTOR-mediated phosphorylation of Ser863 and Ser859 in raptor affect mTORC1 activity as a feedback (46–48). Furthermore, AMPK and p90 ribosomal S6 kinase (RSK) regulate mTORC1 activity via directly phosphorylating Raptor (48). Interestingly, these phosphorylation sites are all found to be conserved in Ss-Raptor, suggesting that Raptor may also regulate mTOR activity through its own phosphorylation in the early vertebrate rockfish.

Tissue expression profile could help us to understand the potential function of Ss-raptor. It has been acknowledged that human Raptor, just like mTOR, is expressed in all the tissue, and with relative higher mRNA level in skeletal muscle, brain, kidney and placenta (10). However, there is few reports revealing the tissue distribution of Raptor in fish species. In present study, mRNA of Ss-Raptor was found to be expressed in all the detected tissues of rockfish, and the expression levels were relative abundant in lymphoid tissues, including blood, spleen, and head-kidney. Consist with mammals, peripheral blood, and spleen are the peripheral immune organs of fish. Majority lymphocytes colonize in the spleen, and recognize the antigens captured by antigen presenting cells in the blood circulation and are subsequently activated. The high expression of Ss-Raptor in peripheral blood and spleen provides a potential regulatory mechanism for its involvement in immune response. Teleost head kidney resembles the bone marrow of mammals, which produces multiple kinds of immune cells. High expression of Ss-Raptor in the head kidney suggests its potential roles to regulate the maturation of various immune cells. However, further study is remained to illustrate the specific mechanism of this expression pattern.

Our results that Ss-Raptor mRNA expression is up-regulated in the innate immune phase after bacterial infection, and blockade of the Raptor/mTORC1 pathway leads to down-regulation of pro-inflammatory factors, suggests that Raptor/mTORC1 pathway is involved in and regulates the innate immune response in rockfish. mTOR pathway has also been reported to promote innate immunity against bacterial infections in mammals. In macrophages, the up-regulation of mTOR phosphorylation and Raptor respond to LPS stimulation, indicates the potential roles of Raptor during innate immune response (49, 50). Moreover, macrophages that deficiency of TSC1, a negative regulator of Raptor/mTORC1 signaling, produce large amounts of pro-inflammatory cytokines, including TNF-α, IL-12 p40, and IL-6 (50). However, there are also some controversial results regarding the regulation of mTOR pathway on innate immunity in mammals. For example, there is evidence suggesting that activation of mTOR leads to a decrease of IL-12p70 secretion in human macrophages (51). In present study, IL-1β and IL-8 were selected as indicators for innate immune response because they are both pro-inflammatory cytokines, which play essential roles in host defense, pathogen resistance, inflammatory cells recruitment, and acute tissue injury (52, 53). In a mouse model, mTORC1 activity is important for production of both cytokines. Activated mTORC1 promotes pro-IL-1β maturation via regulating hexokinase 1 (HK1)-dependent glycolysis (54), and also elicits IL-8 gene expression via the activation of an IRE1-JNK signaling cascade (55). Here, our results confirmed that Raptor/mTORC1 is crucial for the production of pro-inflammatory IL-1β and IL-8 in a fish species. Although in-depth mechanism needs to be further explored, our study provides preliminary evidence for the regulation roles of Raptor/mTORC1 pathway on innate immunity in early vertebrates.

Activation of T lymphocyte requires the interaction of clonotypic TCR with antigen peptide from major histocompatibility complex (MHC) molecules on the surface of antigen presenting cells (56). Accumulating evidence have shown that mTOR plays an important role in the activation of T lymphocytes. As a classic activating signal, TCR signals trigger both mTORC1 and mTORC2, whose activity is highly related to the dose and duration of antigenic peptide (57). In the activated lymphocytes, the phosphorylation levels of AKT, mTOR, S6K, S6 and 4EBP1 were significantly increased, while the phosphorylation levels were inhibited after rapamycin treatment (58, 59). These evidences reveal that, following antigen stimulation, AKT(Ser308)-mTORC1 is an axis located downstream of the TCR, and will in turn phosphorylate the downstream S6K1 and 4EBP1 (60). However, as early vertebrate, the regulatory mechanism of lymphocyte activation is still unclear in teleost. Our studies indicate that the phosphorylation of AKT (Thr308), S6 (Ser240/Ser244), and 4EBP1 (Thsr37/46) are significantly enhanced upon lymphocyte activation, suggests that Raptor/mTORC1 signaling is involved in lymphocyte activation on phosphorylation level.

Following activation, naive T cells rapidly initiate clonal expansion and functional differentiation to eliminate infection. Our results demonstrate that mTORC1 inhibition reduces the number of lymphocytes and the weight of lymphoid organs, suggesting that mTORC1 activity is indispensable for the lymphocyte expansion. Consistent with this, deletion of Raptor in mouse not only seriously impairs antigen-specific T cell activation and proliferation, but also reduces lymphopenia-induced proliferation (61). Analogously, lymphocyte proliferation was also severely inhibited by rapamycin treatment in rat (62). Together with these evidences, our results propose Raptor/mTORC1 axis might serve as an evolutionary conserved signaling to regulate lymphocyte proliferation throughout the vertebrates. When effector lymphocytes traffic to the inflammatory site, they secrete a large amount of pro-inflammatory cytokines such as IL-4, IL-13, IFN-γ, which induce an intense inflammatory response to clear the pathogen infection. Simultaneously, effector CD8^+^ T cells, as cytotoxic T lymphocyte, produce Granzyme B, Perforin A, or bind to Fas on the surface of target cells to lysis infected cells (63). More importantly, these inflammatory or cytotoxic molecules are highly associated with mTOR activity, because rapamycin treatment impairs the inflammatory cell infiltration and pro-inflammatory factors expression (64). Herein, we reported that, during *L. anguillarum* infection, lymphocytes infiltration, as well as mRNA expression of TNF-α, Granzyme B and Perforin A in lymphocytes, were significantly impaired when Raptor/mTORC1 was suppressed by rapamycin. Thus, Raptor/mTORC1 axis regulated effector lymphocytes function is not exclusive for mammalian lymphocytes, but has been emerged in early vertebrate teleost before the divergence of bony fish from the tetrapod lineage.

In summary, we identified an evolutionary conserved Raptor from rockfish, and deduced a Raptor/mTORC1 axis existing in this fish species. Raptor/mTORC1 regulates innate immune response by controlling pro-inflammatory cytokine production, and is involved in adaptive immune responses by regulating lymphocyte activation, proliferation, and infection clearance. Since regulatory mechanism of lymphocyte-mediated immunity is still limited in teleost, our results have provided value evidences to understand the regulation of adaptive immunity in teleost, and thus fill in a gap regarding the evolutionary process of adaptive immune system.

## Data Availability Statement

All datasets generated for this study are included in the article/supplementary material.

## Ethics Statement

All fish care and experimental procedures were performed in accordance with the Guide for the Care and Use of Laboratory Animals of the Ministry of Science and Technology of China, and were approved by the East China Normal University Experimental Animal Ethics Committee. All efforts were made to minimize the suffering of the animals.

## Author Contributions

KL performed experiments, analyzed data and wrote the paper. LZ and HC performed experiments. XW and JY conceived the project, designed experiments, analyzed data, and wrote the paper.

### Conflict of Interest

The authors declare that the research was conducted in the absence of any commercial or financial relationships that could be construed as a potential conflict of interest.
